# COVID-19 and acute or chronic kidney disease: a crescent learning

**DOI:** 10.1590/2175-8239-JBN-2022-E005en

**Published:** 2022-07-29

**Authors:** Cibele Isaac Saad Rodrigues, Rafael Bellotti Azevedo, Elizabeth Silaid Muxfeldt

**Affiliations:** 1 Pontifícia Universidade Católica de São Paulo, Faculdade de Ciências Médicas e da Saúde, Sorocaba, SP, Brazil; 2 Universidade Estácio de Sá, Campus Vista Carioca, Rio de Janeiro, RJ, Brazil; 3 Universidade Federal do Rio de Janeiro, Rio de Janeiro, RJ, Brazil

The COVID-19 pandemic has impacted the world in early 2020. Gradually, we became aware that we were dealing with a multisystemic disease arising from a general inflammatory process that can lead to severe forms and has high levels of morbidity and mortality. Patients hospitalized with symptoms of Severe Acute Respiratory Syndrome (SARS) usually also had their kidneys affected in the form of acute kidney injury (AKI), both in individuals with previously normal kidney function and in patients with previous chronic kidney disease (CKD) who evolved with worsening of their kidney function^1^. Histopathological exams revealed different kinds of lesions - tubular, especially acute tubular necrosis; vascular, with thrombotic microangiopathy; interstitial; and, in some cases, glomerular, notably the collapsing form of glomerulosclerosis, both segmental and focal, compatible with the most frequent clinical and laboratorial presentation of AKI^2,3^. The main factors associated with AKI development are diabetes, hypertension, and/or dyslipidemia. These patients have ah longer hospitalization and a worse prognosis^3^, are more likely to develop cytokine storm, and have a higher mortality^4^. On the other hand, the situation of patients with CKD is also important since they are more vulnerable to severe forms of COVID-19^5^. A recent study showed that these patients have with higher need of renal replacement therapy and their mortality increases proportionally to CKD stages IV and V^6^.

Two Portuguese studies are presented in this JBN issue^7,8^. One study^7^ retrospectively investigated transitory and permanent impacts of AKI in intra-hospital mortality among 544 patients with COVID-19, 330 of which developed AKI based on KDIGO criteria, 166 having the persistent form and 164, the transitory form. The most important risk factors for temporary change of kidney function were age, comorbidities, use of renin angiotensin aldosterone system inhibitors (63.4% were hypertensive), higher creatinine levels, higher acidemia on admission, need of mechanical ventilation, and use of vasopressor drugs. On the other hand, persistent AKI was associated with higher creatinine level (1.71 mg/dL vs 1.25 mg/dL) in hospital admission. Hospital mortality was 14% and higher among patients with AKI (18.5% vs. 7.0%). In multivariate analysis, previous CKD and serum ferritin were independent predictors of AKI. Transient acute kidney injury was not able to predict mortality, but persistent change in kidney function, age, and lactate and ferritin levels were.

The other study retrospectively analyzed 130 patients with CKD^8^, 60% of which were men, with average age of 73.9 years, 81.5% were hypertensive, 54.6% diabetic, and 36.2% had previous cardiovascular disease. Eighty percent of participants developed AKI and 16.2% needed ICU treatment on admission. The 34 patients who died were older and more prone to develop heart failure. Older age, higher ferritin, and higher LDH levels were independent risk factors for mortality.

Although long-term outcomes of AKI associated with COVID-19 have not yet been described, several studies^9,10^ warn of the need for long-term follow-up of patients after hospital discharge, because a significant percentage do not recover their kidney function, and even in those in whom creatinine and estimated glomerular filtration rate have theoretically normalized, there is no assurance that this improvement will persist. This is of concern because some studies have shown a decrease in kidney function after 6 to 12 months of follow-up, even among patients who did not present AKI symptoms during the acute phase^10^.

A systematic review with metanalysis that included 36 studies and 6,395 patients with COVID-19 showed that pre-existing CKD increases the risk of severe disease by more than threefold (OR = 3.28), and that AKI was significantly higher in the group of critical patients than in the severe group (OR=13.92), and also when comparing severe and less severe patients (OR=11.02)^11^.

Actually, it does not matter whether the disease occurs acutely in a patient without previous kidney function deficit, or whether it affects an already chronic patient. Both situations are severe and carry a significant additional risk of morbidity and death. As long-term post-COVID consequences remain unknown, and there are no guidelines for monitoring renal function after an acute infection^10^, public policies must be developed worldwide for the follow-up of patients who needed hospitalization due to severe forms of COVID-19. Standard measures, applied indiscriminately, can reduce the inequality between individuals living in high- and low-income countries.

To date, some answers are not yet clear, as they depend on time of follow-up and well-established protocols that use the same parameters of evaluation and outcomes to allow comparisons. Therefore, it is necessary to develop prospective studies to assess kidney function using several parameters, such as kidney biopsy, urinalysis, direct and indirect measurements of glomerular filtration rate, and tubular function assessment^10^.

Certainly, understanding of the disease and its consequences is increasing worldwide, but new variants have been identified, and we must also consider genetic variability in responses, known risk factors (comorbidities, older age), as well as lesser-known ones, such as race and social vulnerability. It is not yet known how this entangled complex affects different organs and systems, including the kidneys, providing much room for study and learning^10^.
